# Unilateral versus Bilateral Endoscopic Nasobiliary Drainage and Subsequent Metal Stent Placement for Unresectable Malignant Hilar Obstruction: A Multicenter Randomized Controlled Trial

**DOI:** 10.3390/jcm10020206

**Published:** 2021-01-08

**Authors:** Ryunosuke Hakuta, Hirofumi Kogure, Yousuke Nakai, Hiroshi Kawakami, Hiroyuki Maguchi, Tsuyoshi Mukai, Takuji Iwashita, Tomotaka Saito, Osamu Togawa, Saburo Matsubara, Tsuyoshi Hayashi, Iruru Maetani, Yukiko Ito, Osamu Hasebe, Takao Itoi, Keiji Hanada, Hiroyuki Isayama

**Affiliations:** 1 Department of Gastroenterology, Graduate School of Medicine, The University of Tokyo, Tokyo 113-8655, Japan; hakuta-tky@umin.ac.jp (R.H.); kogureh.tky@gmail.com (H.K.); ynakai.tky@gmail.com (Y.N.); tomsaito-gi@umin.ac.jp (T.S.); 2 Department of Endoscopy and Endoscopic Surgery, Graduate School of Medicine, The University of Tokyo, Tokyo 113-8655, Japan; 3 Department of Gastroenterology and Hepatology, Hokkaido University Graduate School of Medicine, Sapporo 060-8648, Japan; hiropon@med.miyazaki-u.ac.jp; 4 Department of Gastroenterology and Hepatology, Faculty of Medicine, University of Miyazaki, Miyazaki 889-1692, Japan; 5 Center for Gastroenterology, Teine-Keijinkai Hospital, Sapporo 006-8555, Japan; maguchi@tb3.so-net.ne.jp (H.M.); thayashi244@gmail.com (T.H.); 6 Department of Gastroenterology, Gifu Municipal Hospital, Gifu 500-8513, Japan; tsuyomukai@yahoo.co.jp; 7 First Department of Internal Medicine, Gifu University Hospital, Gifu 501-1194, Japan; takuji729@gmail.com; 8 Department of Gastroenterology, JR Tokyo General Hospital, Tokyo 151-8528, Japan; 9 Department of Gastroenterology, Kanto Central Hospital, Tokyo 158-8531, Japan; togawao-tky@umin.net; 10 Department of Gastroenterology, Tokyo Metropolitan Police Hospital, Tokyo 164-8541, Japan; saburo.matsubara@gmail.com; 11 Department of Gastroenterology and Hepatology, Saitama Medical Center, Saitama Medical University, Saitama 350-8550, Japan; 12 Department of Medical Oncology and Hematology, Sapporo Medical University School of Medicine, Sapporo 060-8556, Japan; 13 Division of Gastroenterology and Hepatology, Department of Internal Medicine, Toho University Ohashi Medical Center, Tokyo 153-8515, Japan; mtnir50637@med.toho-u.ac.jp; 14 Department of Gastroenterology, Japanese Red Cross Medical Center, Tokyo 150-8935, Japan; yukiko1224md@yahoo.co.jp; 15 Department of Gastroenterology, Nagano Municipal Hospital, Nagano 381-8551, Japan; hasebe@hospital.nagano.nagano.jp; 16 Department of Gastroenterology and Hepatology, Tokyo Medical University, Tokyo 160-0023, Japan; itoi@tokyo-med.ac.jp; 17 Department of Gastroenterology, Onomichi General Hospital, Hiroshima 722-0018, Japan; kh-ajpbd@nifty.com; 18 Department of Gastroenterology, Graduate School of Medicine, Juntendo University, Tokyo 113-8431, Japan

**Keywords:** cholestasis, endoscopic retrograde cholangiopancreatography, endoscopy, jaundice, stents

## Abstract

(1) Background: Endoscopic management of hilar biliary obstruction is still challenging. Compared with unilateral drainage, bilateral drainage could preserve larger functional liver volume and potentially improve clinical outcomes. To evaluate the effectiveness of bilateral drainage, we conducted this multicenter randomized controlled study. (2) Methods: Patients with unresectable malignant hilar biliary obstruction were assigned to unilateral or bilateral group. At first, patients underwent endoscopic nasobiliary drainage (ENBD), and subsequently underwent self-expandable metallic stent (SEMS) deployment. Primary outcomes were the functional success rate of ENBD and time to recurrent biliary obstruction (TRBO) after SEMS deployment. (3) Results: During the study period, 38 and 39 patients were enrolled in the unilateral and bilateral groups. The functional success rate was similar in the uni- and bi-ENBD group (57% vs. 56%; *p* = 0.99), but the rate of additional drainage was higher in uni-ENBD group. Although TRBO and overall survival time after SEMS deployment were not different between the groups (*p* = 0.11 and 0.78, respectively), the incidence of early adverse events tended to be higher in the bi-SEMS group (5.3% vs. 28%; *p* = 0.11). (4) Conclusions: Our study failed to demonstrate the superiority of bilateral over unilateral biliary drainage in terms of functional success rate and TRBO.

## 1. Introduction

Endoscopic retrograde cholangiopancreatography (ERCP) is a widely standardized technique for the treatment of obstructive jaundice [1]. However, endoscopic management of malignant biliary obstruction, especially in patients with hilar stricture, is still challenging even for experienced endoscopists. Among various unmet needs for management of malignant hilar biliary obstruction [2], the superiority of bilateral or unilateral biliary drainage is still under debate [3,4,5,6,7]. Liver volume with successful biliary drainage was reported to be associated with functional success in cases with hilar biliary obstruction [8], thus bilateral drainage has a potential advantage by the preservation of larger functional liver volume compared with unilateral drainage. However, given a possible increased risk of early adverse events after bilateral drainage [7,9,10,11], it is still controversial whether bilateral biliary drainage improves clinical outcomes of malignant hilar biliary obstruction.

Technical difficulty is another concern in bilateral drainage, especially in self-expandable metallic stent (SEMS) deployment. Compared with unilateral drainage, bilateral drainage could be associated with higher technical failure [9,12]. Traditionally, bilateral SEMS deployment for malignant hilar biliary obstruction is not widely accepted because of the complicated endoscopic maneuver. Recently, the improvement of metallic stents allows easy bilateral SEMS deployment [13,14,15,16] but technical success rates of bilateral SEMS deployment still vary among studies [3,4,9]. Furthermore, it is also controversial whether this technical difficulty of bilateral SEMS deployment can lead to better long term outcomes or not. Therefore, we conducted this multicenter prospective two-step randomized controlled study to compare functional success between bilateral and unilateral biliary drainage (Step 1) and recurrent biliary obstruction after bilateral and unilateral SEMS placement (Step 2).

## 2. Material and Methods

### 2.1. Study Design

The current study was a multicenter randomized controlled study to evaluate short- and long-term outcomes of unilateral and bilateral endoscopic biliary drainage for patients with unresectable malignant hilar biliary obstruction. This study was conducted at 16 Japanese academic centers or tertiary care centers. Clinical outcomes were compared between the unilateral and bilateral groups.

This study was conducted according to the Consolidated Standards of Reporting Trials guidelines and was approved by the ethics committee at each institution. Written informed consent was obtained from each patient before the procedure. The study was registered in the University Hospital Medical Information Network clinical trials registry (UMIN000007859).

### 2.2. Patients

We identified patients aged 20 years or older with obstructive jaundice (≥3 mg/dL) due to unresectable malignant hilar biliary obstruction with Bismuth type II, IIIa, IIIb, and IV. Eligible patients were randomly assigned to unilateral or bilateral biliary drainage group prior to ERCP. The exclusion criteria were as follows: (1) patients after gastrectomy with Billroth-II or Roux-en-Y reconstruction, (2) patients who failed scope insertion to the duodenal papilla, (3) patients after hepatectomy, (4) patients with severe cholangitis, and (5) pregnant patients. At first, eligible patients were allocated to unilateral or bilateral group. Step 1 was conducted to evaluate the effectiveness of bilateral biliary drainage. Unilateral or bilateral endoscopic nasobiliary drainage (ENBD) was performed by the allocated group [17,18], and functional success rates were compared between the groups. Subsequently, Step 2 was conducted to evaluate long-term outcomes of unilateral or bilateral SEMS deployment. The following patients subsequently underwent SEMS deployment: (1) patients with improved jaundice (≤50% of total bilirubin level before the drainage) within one week after the initial drainage, (2) patients who underwent additional biliary drainage due to inadequate drainage or cholangitis, and improved within two weeks. Patients who were allocated to the unilateral group but underwent bilateral SEMS deployment after additional drainage were analyzed as bilateral SEMS (bi-SEMS) group. On the contrary, patients who were allocated to the bilateral group but underwent unilateral SEMS deployment due to technical failure of bilateral ENBD placement were analyzed as unilateral SEMS (uni-SEMS) group. Time to recurrent biliary obstruction (TRBO) was compared between the bi-SEMS and uni-SEMS groups.

The primary outcomes of this study were the functional success rate in Step 1 and TRBO in Step 2. The secondary outcomes of Step 1 included technical success rate of ENBD placement, bilirubin decrease rate, the incidence of early adverse events associated with ENBD placement, and the rate of additional biliary drainage. Secondary outcomes of Step 2 included technical success rate of SEMS placement, early and late (within or after 30-day of the index ERCP of SEMS placement) adverse events associated with SEMS deployment, overall survival time, and the rate of successful re-intervention for recurrent biliary obstruction.

### 2.3. Definitions of Outcome Variables

The definitions of technical and functional success were successful endoscopic biliary drainage for preprocedural scheduled biliary tree and reduction in serum total bilirubin level under 50% before biliary drainage within one week of the index ERCP, respectively. Recurrent biliary obstruction was defined as an elevated liver enzyme (total serum bilirubin level >3.0 mg/dL, alkaline phosphatase or γ-glutamyl transpeptidase >3-times upper limit of the normal range) or cholangitis which required endoscopic treatment [19]. The other adverse events were defined according to the lexicon guidelines proposed by the American Society of Gastrointestinal Endoscopy [20].

### 2.4. Sample Size Calculation

The functional success rate was hypothesized as 75% in the unilateral ENBD (uni-ENBD) group compared to 90% in the bilateral ENBD (bi-ENBD) group in Step 1. To estimate the superiority of the bilateral biliary drainage group, 79 patients per group were required (α 0.05, power 80%). Considering 5% of ineligible cases, an appropriate sample size was calculated as 83 patients in each group.

### 2.5. Randomization

Randomization was performed centrally before ERCP using a minimization computer algorithm, stratified by primary cancer, Bismuth type, and centers. Eligible patients were randomized in a 1:1 ratio to the unilateral or bilateral group. The allocation was not masked for either patients or investigators. Modified intention-to-treat analysis was performed after excluding those patients who did not undergo ERCP.

### 2.6. Endoscopic Procedures

The drainage area in the unilateral or bilateral groups was selected before the procedure using computed tomography (CT) or magnetic resonance cholangiopancreatography (MRCP).

A duodenoscope was inserted under moderate sedation using intravenous injection of diazepam or midazolam and pethidine hydrochloride. Wire-guided cannulation or contrast-assisted cannulation was used for biliary cannulation at the discretion of the endoscopists. After a guidewire was placed into the preplanned bile duct, cholangiogram was achieved for confirmation. In Step 1, unilateral or bilateral ENBD was placed using a 5-F or 6-F catheter. No patients underwent plastic stent or SEMS placement in the initial session. 

**Figure 1:**
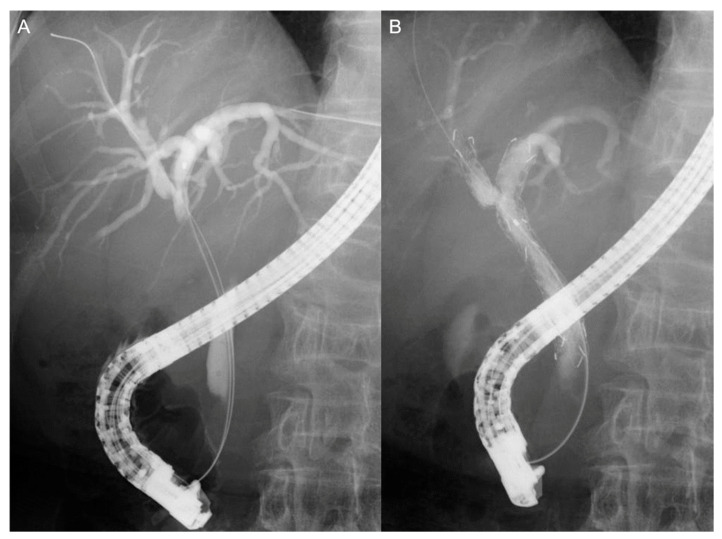
Fluoroscopic images of endoscopic biliary drainage for hilar biliary obstruction. (**A**) Cholangiogram in a patient with hilar cholangiocarcinoma. (**B**) uncovered self-expandable metallic stents were deployed in a stent-in-stent fashion.

Patients with successful resolution of jaundice or cholangitis by the placement of ENBD subsequently proceeded to Step 2 and underwent SEMS placement. Modified Niti-S large cell D-type stent (LCD; Taewoong Corp., Seoul, Korea) in diameter of 8 or 10 mm and length of 6 or 8 cm was used [15]. LCD has a large cell size (7 mm) and 8-F delivery system facilitating stent-in-stent deployment. SEMS was deployed to the same biliary duct where ENBD had been already placed. In the bilateral group, SEMS was placed in a stent-in-stent fashion (Figure 1).

### 2.7. Statistical Analysis

Categorical variables were compared using the chi-square test or Fisher’s exact test, as appropriate. Continuous variables were compared using the Wilcoxon rank-sum test. TRBO and overall survival time were estimated using the Kaplan-Meier method and compared using the log-rank test between the groups. Uni- and multi-variable logistic regression models were used to estimate odds ratios and corresponding 95% confidence interval (CI) of functional success in Step 1. The multivariable model included variables with a *p* value of <0.20 in a univariable model in addition to the drainage group (unilateral vs. bilateral).

As previously reported [21], decreasing bilirubin rate after unilateral or bilateral ENBD placement was estimated using b value calculated by the nonlinear least-squares method fitted to the equation y = ae^bx^. In this equation, y is the serum bilirubin level, x is the number of days after biliary drainage, and a is represented bilirubin levels on the drainage day. 

For all analyses, a two-sided *p* value < 0.05 was used to denote statistical significance. All statistical analyses were performed using the EZR software (Saitama Medical Center, Jichi Medical University, Saitama, Japan), which is a graphical user interface for the R software (The R Foundation for Statistical Computing, Vienna, Austria, version 3.4.1) [22].

## 3. Results

**Figure 2:**
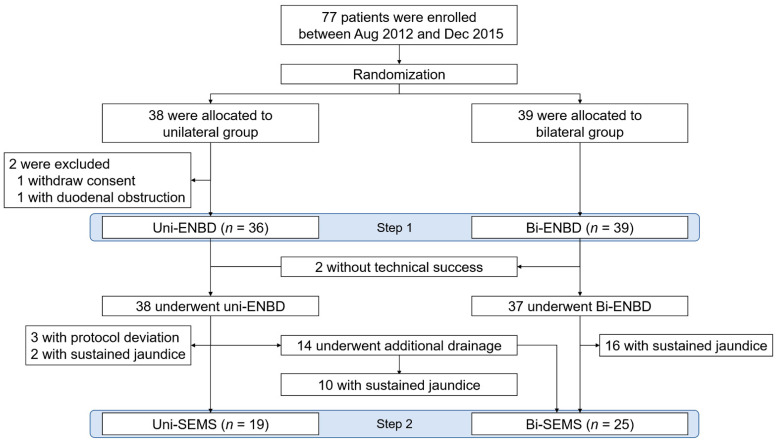
Flowchart of selection into uni- and bi-lateral endoscopic nasobiliary drainage group for patients with unresectable malignant hilar biliary obstruction. Bi-ENBD, bilateral endoscopic nasobiliary drainage; Bi-SEMS, bilateral self-expandable metallic stent; ENBD, endoscopic nasobiliary drainage; SEMS, self-expandable metallic stent; Uni-ENBD, unilateral endoscopic nasobiliary drainage; Uni-SEMS, unilateral self-expandable metallic stent.

The current study was conducted between August 2012 and December 2015, and a total of 77 patients were enrolled for this study (Figure 2). After the randomization, 38 patients were allocated to the unilateral group and 39 patients to the bilateral group. The study was terminated prematurely due to poor patient enrollment. 

**Figure 3:**
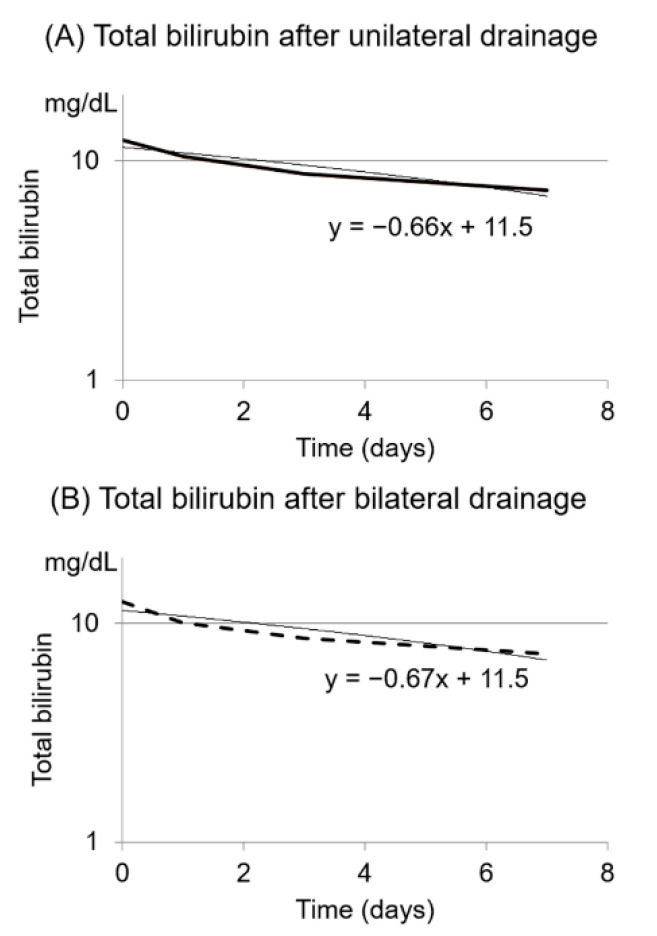
Bilirubin decrease rates after unilateral (**A**) and bilateral (**B**) endoscopic nasobiliary drainage. The serum total bilirubin level was log-transformed, and bilirubin decrease rate was estimated using the nonlinear least-squares method (Step 1).

Baseline characteristics including primary cancer and Bismuth type were well-balanced between the groups (Table 1). Table 2 shows the clinical outcomes of uni- and bi-ENBD groups (Step 1). Two patients who did not undergo ERCP in the unilateral group were excluded from analyses of Step 1. The technical success rate of ENBD placement was high in both groups (100% vs. 95% in the uni-ENBD and bi-ENBD group; *p* = 0.49) but the rate of functional success was 57% in the uni-ENBD and 56% in the bi-ENBD group, respectively (*p* = 0.99). Drainage group (uni-ENBD vs. bi-ENBD) was not associated with functional success in the multivariable logistic regression model; multivariable-adjusted odds ratio of the bi-ENBD group compared with the uni-ENBD group was 1.11 with 95% confidence interval of 0.42–2.95 (*p* = 0.83; Table 3 and Table S1). Bilirubin decreasing rates were similar between the groups; the b value was −0.60 in the unilateral group and −0.61 in the bilateral group, respectively (Figure 3). Additional biliary drainage was performed for 14 patients (39%) in the uni-ENBD group and two (5.3%) in the bi-ENBD group (*p* < 0.001). The incidence of early adverse events associated with ENBD placement did not have a statistically significant difference between the two groups (19% vs. 31%, in the uni- and bi-ENBD groups, respectively; *p* = 0.30).

**Figure 4:**
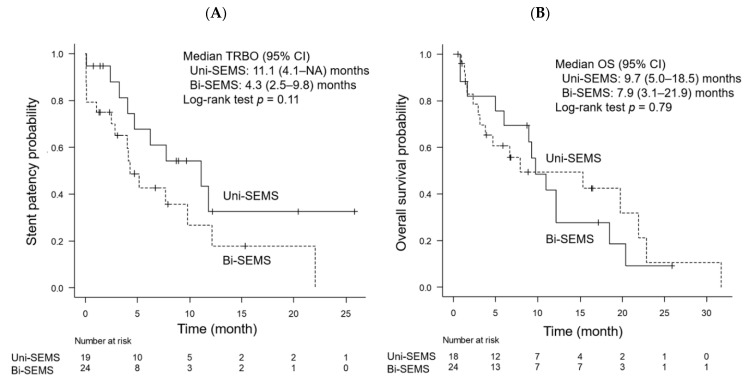
Kaplan–Meier curve of recurrent biliary obstruction (**A**) and overall survival (**B**) in the uni- and bi-SEMS group. *p* values were calculated using the log-rank test (Step 2). Bi-SEMS, bilateral self-expandable metallic stent; CI, confidence interval; NA, not available; OS, overall survival; SEMS, self-expandable metallic stent; TRBO, time to recurrent biliary obstruction; Uni-SEMS, unilateral self-expandable metallic stent.

Patients who successfully improved jaundice or cholangitis subsequently underwent uni- or bi-SEMS deployment (Step 2). After excluding 28 patients with sustained jaundice and 3 with protocol deviation, 19 and 25 patients underwent uni- and bi-SEMS deployment. The diameter of SEMS was 8 and 10 mm in one (5.3%) and 18 patients (95%) in the uni-SEMS group compared with four (16%) and 21 patients (84%) in the bi-SEMS group, respectively (*p* = 0.37). Figure 4 shows Kaplan–Meier curves of recurrent biliary obstruction and overall survival time in the uni- and bi-SEMS groups. Median TRBO was 11.1 months in the uni-SEMS group, and 4.3 months in the bi-SEMS group, but between-group difference was not observed between the groups (*p* = 0.11). In addition, neither overall survival time was different between the groups (*p* = 0.78). SEMS deployment was technically successful in all patients in the uni- and bi-SEMS group. The incidence of early adverse events was 5.3% in the uni-SEMS group and 28% in the bi-SEMS group (*p* = 0.11), respectively (Table 4). The majority of early adverse events associated with bi-SEMS deployment was cholangitis. The incidence of late adverse events was similar between the groups (47% vs. 44%, in the uni- and bi-SEMS group, respectively; *p* = 0.99). Reintervention for recurrent biliary obstruction was successful in nine among ten patients (90%) of the uni-SEMS group and 13 among 14 patients (93%) of the bi-SEMS group (*p* = 0.99).

## 4. Discussion

The current study was a randomized controlled trial to evaluate the superiority of the bilateral endoscopic biliary drainage compared with unilateral drainage for patients with unresectable malignant hilar biliary obstruction. Compared with the unilateral group, the bilateral group showed neither better functional success rate after ENBD (Step 1) nor improved TRBO and overall survival time after SEMS deployment (Step 2). The incidence of additional drainage after initial ENBD placement was higher in the unilateral group. However, the rate of early adverse events associated with SEMS deployment tended to be higher in the bilateral group.

The rate of functional success was not different between the bilateral and unilateral ENBD group (Step 1) but was unexpectedly lower compared with our hypothesis and previous studies [3,23,24,25]. This discrepancy was thought to be derived from the differences in the definition of functional success between the studies. In the current study, functional success was judged within one week of the index ERCP due to the discomfort by prolonged ENBD placement, whereas most of the reports defined functional success within one month [3,24,26]. Due to this limited duration of time in our study, the rate of functional success might be lower than the hypothesis.

A previous study suggested the association between functional success and liver volume with successful biliary drainage in patients with hilar biliary obstruction [8] and recommended to undergo biliary drainage more than 50% of the total liver volume. Compared with bilateral drainage, unilateral drainage could preserve only limited functional liver volume. Consistent with previous studies [8,12], the current study also suggested the higher incidence of additional drainage after unilateral drainage due to inadequate jaundice resolution. Considering the comparable safety of bilateral and unilateral ENBD, bilateral biliary drainage was thought to be a reasonable option for malignant hilar biliary obstruction.

Bilateral SEMS deployment did not improve TRBO and overall survival time in Step 2 of our study. A recent randomized controlled trial comparing the outcome of bilateral and unilateral SEMS deployment showed longer TRBO after bilateral drainage: 252 days in the bi-SEMS group and 139 days in the uni-SEMS group, respectively (*p* < 0.01) [3]. Contrary to this study, our study included not only patients with biliary tract cancer but also metastases to the hilum from cancers in other organs. Patients with metastatic cancer have poor general status, and inclusion of these patients could offset the potential benefit of bilateral SEMS deployment [26]. In addition, the previous randomized study consecutively used BONASTENT M-Hilar (Standard Sci Tech Inc, Seoul, South Korea), and the difference in the stent design might affect the study outcomes including stent patency. Furthermore, compared to unilateral SEMS, the incidence of early adverse events associated with bilateral SEMS tended to be higher in our study. Clinical evidence suggested an increased incidence of adverse events after bilateral drainage including cholangitis or liver abscess [7,9,10]. Complicated endoscopic procedure during bilateral SEMS deployment potentially increases early adverse events through unintended cholangiogram to biliary trees without drainage. Although comparable safety of bilateral and unilateral SEMS deployment was also reported [3,4], further studies are needed to elucidate the patient cohort who could benefit from bilateral SEMS deployment. Considering the longer tendency of TRBO in uni-SEMS group, patients with successful functional success by unilateral predrainage procedure could avoid bilateral SEMS deployment.

A multicenter randomized design was the major strength of our study. The current study was conducted in five academic hospitals and 11 tertiary care centers in Japan with experts of ERCP and showed a high technical success rate in both unilateral and bilateral endoscopic biliary drainage. There were several limitations to our study. At first, we only included patients of unresectable malignant hilar biliary obstruction at the time of initial drainage. Due to the difficulty in diagnosing patients as unresectable only by local disease extension in imaging studies prior to ERCP, the majority of patients had distant metastasis in our study. In clinical practice, the local disease extension is evaluated by ERCP and adjunctive procedures such as intraductal ultrasonography or cholangioscopy in addition to CT and MRCP. Furthermore, the diagnostic difficulty hampered smooth patient recruitment and resulted in premature termination of the study. Secondly, patients with additional drainage after uni-ENBD placement were included in the bi-SEMS group. Due to the two-step approach in our study, bi-SEMS group potentially included patients with advanced tumor status and our study may fail to show the effectiveness of bilateral SEMS deployment. Third, we calculated the sample size to compare the functional success rate of bilateral and unilateral ENBD. Therefore, this study setting was not adequate to evaluate long-term outcomes after bilateral and unilateral SEMS deployment. Finally, new approaches to hilar malignant biliary obstruction have been increasingly reported such as intraductal ablation therapy [27] and combined ERCP and endoscopic ultrasonography-guided biliary drainage [28] and should be further investigated in prospective comparative studies.

In summary, bilateral endoscopic biliary drainage was not associated with better functional success rate or longer TRBO compared with unilateral drainage. Further study is needed to investigate the cohort of patients who benefit from bilateral SEMS deployment.

## Figures and Tables

**Table 1:** Baseline characteristics of patients with malignant hilar biliary obstruction who were allocated to unilateral or bilateral biliary drainage group.

Characteristic *	Unilateral (*n* = 38)	Bilateral (*n* = 39)	*p* Value
Age	78 (72–82)	72 (69–81)	0.15
Gender			0.24
Male	21 (55%)	27 (69%)	
Female	17 (45%)	12 (31%)	
ASA-PS score ^†^, 1/2/3	15/18/5 (39%/47%/13%)	17/22/0 (44%/56%/0%)	0.07
Primary cancer			0.89
Hilar cholangiocarcinoma	12 (32%)	13 (33%)	
Gallbladder cancer	10 (26%)	12 (31%)	
ICC	6 (16%)	6 (15%)	
Others	10 (26%)	8 (21%)	
Bismuth type			0.92
II	10 (26%)	10 (26%)	
IIIa	11 (29%)	9 (23%)	
IIIb	2 (5.3%)	2 (5.1%)	
IV	15 (40%)	18 (46%)	
Liver metastasis	12 (32%)	16 (41%)	0.48
Ascites	7 (18%)	7 (18%)	0.99
Previous gastrectomy	1 (2.6%)	1 (2.6%)	0.99
Laboratory data			
White blood cell, 10^4^/μL	6.1 (5.3–7.3)	6.5 (5.0–8.2)	0.33
CRP, mg/dL	1.4 (0.6–3.2)	1.5 (0.8–3.4)	0.68
Albumin, mg/dL	3.2 (2.8–3.7)	3.3 (2.9–3.5)	0.82
Total bilirubin, mg/dL	10 (5.1–15)	9.7 (5.7–17)	0.88
Prothrombin time, s	95 (82–95)	93 (75–93)	0.50

* Data are expressed as number (percentage) of patients within a given group or as median (interquartile range). ^†^ ASA-PS score is defined as follows: 1, a normal healthy patient; 2, a patient with a mild systemic disease; 3, a patient with a severe systemic disease. ASA-PS, American Society of Anesthesiologists Physical Status Classification; ICC, intrahepatic cholangiocarcinoma.

**Table 2:** Clinical outcomes of patients who underwent unilateral or bilateral endoscopic nasobiliary drainage (Step 1).

Outcome *	Uni-ENBD (*n* = 36)	Bi-ENBD (*n* = 39)	*p* Value
Technical success	36 (100%)	37 (95%)	0.49
Functional success	21 (57%)	22 (56%)	0.99
Additional drainage	14 (39%)	2 (5.3%)	<0.001
ENBD	12 (33%)	2 (5.1%)	
PTBD	1 (2.8%)	0	
Plastic stent	1 (2.8%)	0	
Early adverse events	7 (19%)	12 (31%)	0.30
Cholangitis	4 (11%) ^†^	4 (10%)	0.99
Cholecystitis	0	1 (2.6%)	0.99
Pancreatitis	4 (11%) ^†^	3 (7.7%)	0.70
Liver abscess	0	1 (2.6%)	0.99
Gastrointestinal bleeding	0	2 (5.1%)	0.49
Self-removal of ENBD	0	1 (2.6%)	0.99

* Data are expressed as number (percentage) of patients within a given group. Bi-ENBD, bilateral endoscopic nasobiliary drainage; ENBD, endoscopic nasobiliary drainage; PTBD, percutaneous transhepatic biliary drainage; Uni-ENBD, unilateral endoscopic nasobiliary drainage. ^†^ One patient in the unilateral group developed both cholangitis and pancreatitis.

**Table 3:** Multi-variable logistic regression analyses to assess the factors associated with functional success after endoscopic nasobiliary drainage for malignant hilar biliary obstruction (Step 1).

Subgroup	Total, *n*	FS	OR (95% CI)			
			Univariable	*p* Value	Multivariable *	*p* Value
Drainage						
Uni-ENBD	36	21 (58%)	1 (referent)		1 (referent)	
Bi-ENBD	39	22 (56%)	0.92 (0.37–2.31)	0.87	1.11 (0.42–2.95)	0.83

* To select variables for the final multivariable model, a univariable logistic regression model was examined for each of the following variables: Drainage (unilateral or bilateral), age (<75 or ≥75 years), gender (female or male), American Society of Anesthesiologists Physical Status Classification score (<2 or ≥2), Primary disease (hilar cholangiocarcinoma or others), total bilirubin (<10 or ≥10 mg/dL), albumin (<3.0 or ≥3.0 mg/dL), Bismuth type (IV or others), liver metastasis (yes or no), and ascites (yes or no). In addition to drainage (unilateral or bilateral), variables with *p* value < 0.20 in univariable analyses were entered into the multivariable model. The final model was described in Table S1. Bi-ENBD, bilateral endoscopic nasobiliary drainage; CI, confidence interval; ENBD, endoscopic nasobiliary drainage; FS, functional success; OR, odds ratio; Uni-ENBD, unilateral endoscopic nasobiliary drainage.

**Table 4:** Clinical outcomes of uni- or bi-lateral self-expandable metallic stent placement for patients with malignant hilar biliary obstruction (Step 2).

Outcomes *	Uni-SEMS (*n* = 19)	Bi-SEMS (*n* = 25)	*p* Value
Technical success	19 (100%)	25 (100%)	0.99
Early adverse events	1 (5.3%)	7 (28%)	0.11
Cholangitis	1 (5.3%) ^†^	5 (20%) ^†^	0.21
Pancreatitis	0	1 (4.0%)	0.99
Acute obstruction	1 (5.3%) ^†^	2 (8.0%) ^†^	0.99
Late adverse events	9 (47%)	11 (44%)	0.99
Recurrent biliary obstruction	8 (42%)	11 (44%)	0.99
Tumor ingrowth	6 (32%)	6 (24%)	0.74
Sludge	2 (11%)	1 (4.0%)	0.57
Tumor bleeding	0	2 (8.0%)	0.50
Cholangitis	0	2 (8.0%)	0.50
Liver abscess	1 (5.3%)	1 (4.0%) ^‡^	0.99
Intra-abdominal abscess	0	1 (4.0%) ^§^	0.99
Median TRBO, month	11.1	4.3	0.11

* Data are expressed as number (percentage) of patients within a given group. Bi-SEMS, bilateral self-expandable metallic stent; SEMS, self-expandable metallic stent; TRBO time to recurrent biliary obstruction; Uni-SEMS, unilateral self-expandable metallic stent. ^†^ One patient in each group developed both cholangitis and acute obstruction. ^‡^ One patient in the bi-SEMS group developed both liver abscess and recurrent biliary obstruction. ^§^ One patient in the bi-SEMS group developed both intra-abdominal abscess and recurrent biliary obstruction.

## Data Availability

Data sharing not applicable.

## Supplementary Materials

The following are available online at https://www.mdpi.com/2077-0383/10/2/206/s1, Table S1: Uni- and multi-variable logistic regression analyses to assess the factors associated with functional success after endoscopic nasobiliary drainage for malignant hilar biliary obstruction (Step 1).
