# Supplementary material for: Unilateral versus Bilateral Endoscopic Nasobiliary Drainage and Subsequent Metal Stent Placement for Unresectable Malignant Hilar Obstruction: A Multicenter Randomized Controlled Trial
Source: J Clin Med. 2021 Jan 8;10(2):206. doi: 10.3390/jcm10020206 (PMC7827318; doi:10.3390/jcm10020206)
Supplement: Supplementary file 1 [file jcm-10-00206-s001.zip › Supplementary table S1.docx]

**Supplementary Table 1.** Uni- and multi-variable logistic regression analyses to assess the factors associated with functional success after endoscopic nasobiliary drainage for malignant hilar biliary obstruction (Step 1).

|  |  |  | OR (95% CI) | | | |
| --- | --- | --- | --- | --- | --- | --- |
| **Subgroup** | Total,  n | FS,  n (%) | Univariable | *P*  value | Multivariable* | *P*  value |
| Drainage |  |  |  |  |  |  |
| Uni-ENBD | 36 | 21 (58%) | 1 (referent) |  | 1 (referent) |  |
| Bi-ENBD | 39 | 22 (56%) | 0.92 (0.37-2.31) | 0.87 | 1.11 (0.42-2.95) | 0.83 |
| Age |  |  |  |  |  |  |
| <75 years | 35 | 22 (63%) | 1 (referent) |  |  |  |
| ≥75 years | 40 | 21 (53%) | 0.65 (0.26-1.65) | 0.37 |  |  |
| Gender |  |  |  |  |  |  |
| Female | 29 | 18 (62%) | 1 (referent) |  |  |  |
| Male | 46 | 25 (54%) | 0.73 (0.28-1.88) | 0.51 |  |  |
| ASA-PS |  |  |  |  |  |  |
| <2 | 32 | 19 (59%) | 1 (referent) |  |  |  |
| ≥2 | 43 | 24 (56%) | 0.86 (0.34-2.18) | 0.76 |  |  |
| Primary disease |  |  |  |  |  |  |
| Hilar cholangiocarcinoma | 23 | 16 (70%) | 1 (referent) |  | 1 (referent) |  |
| Others | 52 | 27 (52%) | 0.45 (0.16-1.28) | 0.13 | 0.50 (0.16-1.55) | 0.23 |
| Total bilirubin |  |  |  |  |  |  |
| <10 mg/dL | 39 | 25 (64%) | 1 (referent) |  |  |  |
| ≥10 mg/dL | 36 | 18 (50%) | 0.56 (0.22-1.41) | 0.22 |  |  |
| Albumin |  |  |  |  |  |  |
| <3.0 mg/dL | 23 | 12 (52%) | 1 (referent) |  |  |  |
| ≥3.0 mg/dL | 52 | 31 (60%) | 1.35 (0.50-3.63) | 0.55 |  |  |
| Bismuth type |  |  |  |  |  |  |
| II, IIIa, or IIIb | 43 | 28 (65%) | 1 (referent) |  | 1 (referent) |  |
| IV | 32 | 15 (47%) | 0.47 (0.19-1.20) | 0.12 | 0.42 (0.16-1.14) | 0.09 |
| Liver metastasis |  |  |  |  |  |  |
| No | 47 | 32 (68%) | 1 (referent) |  | 1 (referent) |  |
| Yes | 28 | 11 (39%) | 0.30 (0.11-0.80) | 0.02 | 0.39 (0.14-1.10) | 0.07 |
| Ascites |  |  |  |  |  |  |
| No | 62 | 35 (57%) | 1 (referent) |  |  |  |
| Yes | 13 | 8 (62%) | 1.23 (0.36-4.20) | 0.74 |  |  |

* Variables with *P* value <0.20 in univariable analyses were entered into the multivariable model.

ASA-PS, American Society of Anesthesiologists Physical Status Classification; Bi-ENBD, bilateral endoscopic nasobiliary drainage; CI, confidence interval; ENBD, endoscopic nasobiliary drainage; ICC, intrahepatic cholangiocarcinoma; FS, functional success; OR, odds ratio; Uni-ENBD, unilateral endoscopic nasobiliary drainage.
